# Genomic Analysis Reveals That Isolation Temperature on Selective Media Introduces Genetic Variation in *Campylobacter jejuni* from Bovine Feces

**DOI:** 10.3390/pathogens11060678

**Published:** 2022-06-12

**Authors:** Sicun Fan, Derek Foster, Shaohua Zhao, Sampa Mukherjee, Yesha Shrestha, Cameron Parsons, Sophia Kathariou

**Affiliations:** 1 Department of Food, Bioprocessing and Nutrition Sciences, North Carolina State University, Raleigh, NC 27695, USA; sfan3@ncsu.edu (S.F.); ctparson@ncsu.edu (C.P.); 2 Department of Population Health and Pathobiology, College of Veterinary Medicine, North Carolina State University, Raleigh, NC 27695, USA; dmfoster@ncsu.edu; 3 U.S. Food and Drug Administration, Center for Veterinary Medicine, Laurel, MD 20708, USA; shaohua.zhao@fda.hhs.gov (S.Z.); sampa.mukherjee@fda.hhs.gov (S.M.); yesha.shrestha@fda.hhs.gov (Y.S.)

**Keywords:** ceftiofur, temperature, cattle, *Campylobacter jejuni*, whole genome sequencing, cgMLST, wgMLST, genetic variation

## Abstract

*Campylobacter jejuni* is commonly isolated on selective media following incubation at 37 °C or 42 °C, but the impact of these temperatures on genome variation remains unclear. Previously, *Campylobacter* selective enrichments from the feces of steers before and after ceftiofur treatment were plated on selective agar media and incubated at either 37 °C or 42 °C. Here, we analyzed the whole genome sequence of *C. jejuni* strains of the same multilocus sequence typing (MLST)-based sequence type (ST) and isolated from the same sample upon incubation at both temperatures. Four such strain pairs (one ST8221 and three ST8567) were analyzed using core genome and whole genome MLST (cgMLST, wgMLST). Among the 1970 wgMLST loci, 7–25 varied within each pair. In all but one of the pairs more (1.7–8.5 fold) new alleles were found at 42 °C. Most frameshift, nonsense, or start-loss mutations were also found at 42 °C. Variable loci CAMP0575, CAMP0912, and CAMP0913 in both STs may regularly respond to different temperatures. Furthermore, frameshifts in four variable loci in ST8567 occurred at multiple time points, suggesting a persistent impact of temperature. These findings suggest that the temperature of isolation may impact the sequence of several loci in *C*. *jejuni* from cattle.

## 1. Introduction

*Campylobacter* spp. (family *Campylobacteraceae*) are Gram-negative, motile, and obligative-microaerophilic bacteria that are leading bacterial foodborne pathogens in the United States and other nations [1,2]. The species primarily responsible for *Campylobacter* infections in humans (campylobacteriosis) is *Campylobacter jejuni* [1], which is typically grown at 37 °C or 42 °C, with 42 °C being the optimal growth temperature [2]. Campylobacteriosis commonly causes acute gastroenteritis but may also lead to the severe autoimmune disease Guillain-Barré syndrome and other complications [3]. The leading sources of transmission to humans include raw or undercooked poultry, raw milk, untreated water, and direct contact with animal feces [4]. During the cycle of infection, *Campylobacter* regulates its gene expression in response to different temperatures, including 42 °C and 37 °C when colonizing the gastrointestinal tract of chickens and humans, respectively, and 4 °C when contaminating refrigerated food products such as raw poultry products and milk [3,5].

Ceftiofur belongs to the 3rd-generation cephalosporins and is used widely to treat respiratory disease in feedlot cattle and postpartum metritis in cows [6,7,8]. In a previous study regarding the impact of ceftiofur on *Campylobacter* in cattle, fecal samples from steers that were treated with ceftiofur crystalline-free acid (CCFA) were enriched in Bolton broth at 37 °C and then incubated on the selective medium modified charcoal-cefoperazone-deoxycholate agar (mCCDA) at both 37 °C and 42 °C [9]. The estimated prevalence of *C. jejuni* was found to be significantly higher at 42 °C than 37 °C [9].

Several studies have reported different impacts of 37 °C and 42 °C on gene expression, involving motility, membrane modification, stress response, and virulence of *C. jejuni* [5,10,11,12], as well as temperature-responsive proteins related to virulence, stress response, and metabolism [12,13]. However, we lack information about the potential impacts of temperature on genome variation of *C*. *jejuni* upon recovery from bovine fecal samples on selective media incubated at 37 °C vs. 42 °C. Sequence variation that may accrue in response to differences in cultivation temperature on the selective media may compromise the use of whole genome sequence (WGS) data for molecular epidemiology investigations and eventual design of effective food safety interventions for *Campylobacter* in bovine reservoirs. In this study, our objective was to identify such possible impacts of temperature on genome sequence content by comparing the WGS data of *C*. *jejuni* strains of the same sequence type (ST) and obtained from the same sample following selective enrichment and plating mCCDA at 37 °C and 42 °C. A panel of bioinformatic tools including core genome multilocus sequence typing (cgMLST) and whole genome multilocus sequence typing (wgMLST) were employed for comparative genomic analysis to assess allelic variation in the genome of these strains.

## 2. Results and Discussion

### 2.1. C. jejuni Strains and Quality of WGS

The 18 *C. jejuni* strains that were selected to examine the potential temperature impacts on the genomes were chosen so as to include strains of the same ST and isolated on mCCDA at different temperatures, i.e., 37 °C and 42 °C. The strain panel included one strain pair of ST8221 and three strain pairs of ST8567. For each of these pairs, one strain was isolated on mCCDA incubated at 37 °C while the other strain was from the same fecal sample following incubation on mCCDA at 42 °C (Table 1). In addition, we included five unpaired ST8221 strains (three from 37 °C and two from 42 °C), and five unpaired strains of ST21, including four from 37 °C and one from 42 °C (Table 1). The assembly of the 18 strains had a median sequence size of 1,719,404 bp, with a mean of 1,712,470.7 (ranging from 1,691,028 to 1,729,077). The median number of contigs was 39.5, with a mean of 49.4 (ranging from 27 to 195) (Table S1). The median N50 value was 121,978.5 bp with a mean of 136,899.8 bp (ranging from 70,661 to 288,016) (Table S1). The quality of the WGS was deemed sufficient, as almost all assembly parameters of the genomes in this study were in the same range as other SPAdes-assembled *C. jejuni* genomes [14,15]. The remaining parameters, i.e., number of reads, average read length, mean contig sequence size, and L50 are summarized in Table S1.

### 2.2. Variable Loci at the Different Temperatures Were Identified in Both STs, with Overall Higher Prevalence of Variable New Alleles at 42 °C Than 37 °C

In both ST8221 and ST8567, an estimated 0.6% of the loci had missing and incomplete alleles in the cgMLST scheme (Table S2). As over 97.3% of the cgMLST loci were detected in both ST8221 and ST8567, the quality of the genome assemblies was considered sufficient for wgMLST analysis [16]. The percentage of missing and incomplete alleles in the cgMLST scheme in both STs was similar to that observed among *C. jejuni* strains of ST45 recovered at different time points from an immunodeficient patient with chronic campylobacteriosis (approx. 0.6% vs. 0.2%) [17].

**Figure 1:**
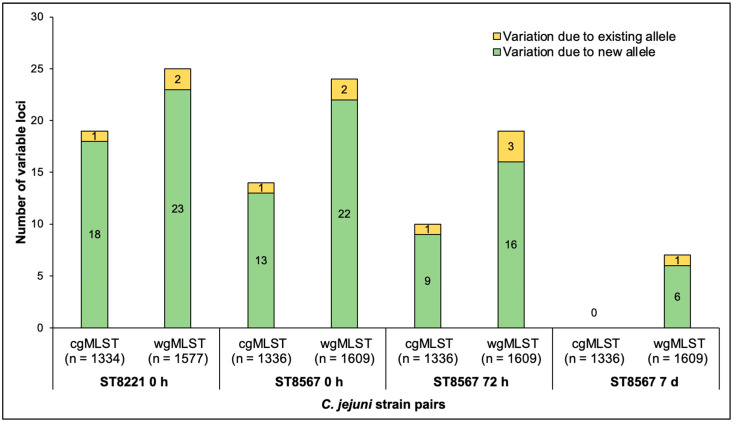
Number of variable loci between paired *C. jejuni* strains of ST8221 and ST8567 isolated at 37 °C and 42 °C. The four strain pairs are, from left to right, ST8221 at 0 h followed by ST8567 at 0 h, 72 h, and 7 d. Yellow and green indicate the variation due to existing and new alleles, respectively. Analysis via cgMLST and wgMLST was carried out as described in Materials and Methods.

After excluding loci with missing and incomplete alleles, 19 cgMLST and 25 wgMLST loci (around 1.5% of the total in each scheme) were found to vary between the two temperatures in the ST8221 strain pair (Figure 1 and Table S3). Similar numbers of variable loci were observed in the ST8567 strain pairs obtained at 0 and 72 h (Figure 1). The third ST8567 strain pair was recovered at 7 d and was unusual in exhibiting noticeably fewer variable loci: only seven wgMLST loci were found to be variable, while none were detected with cgMLST (Figure 1 and Table S3). The number of variable loci was consistently higher in the wgMLST than the cgMLST scheme (Figure 1 and Table S3), as expected based on the larger number of genes in the wgMLST scheme.

**Figure 2:**
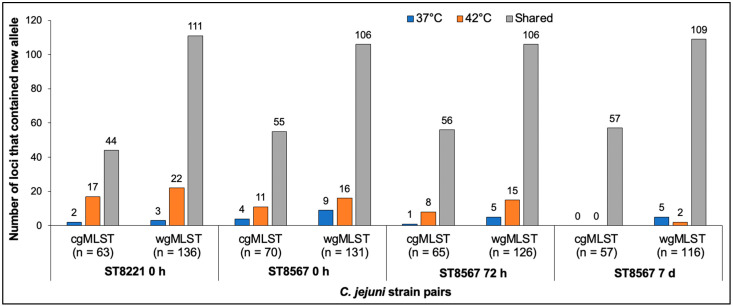
Incidence of loci with new alleles in *C. jejuni* strains isolated at 37 °C and 42 °C. The four strain pairs are, from left to right, ST8221 at 0 h followed by ST8567 at 0 h, 72 h and 7 d. Blue and orange indicate loci with new alleles observed at 37 °C and 42 °C, respectively, while grey indicates loci with the same new alleles at 37 °C and 42 °C. Analysis via cgMLST and wgMLST was carried out as described in Materials and Methods.

Most (84.2–94.7%) of the observed variation for all strain pairs was due to new alleles, i.e., alleles which were not previously encountered in the PubMLST database (Figure 1). Variation involving previously known alleles was found to contribute only 1–3 variable alleles in each paired wgMLST comparison (Figure 1). Analysis of the wgMLST loci with new alleles in the four pairs revealed that most were shared between the members of each pair and the numbers of such shared new alleles were similar among the pairs, ranging from 106 to 111, while a lower number (7 to 25) of wgMLST loci had new alleles that varied between the members of each pair (Figure 2). Interestingly, in three of the four strain pairs (the ST8221 pair and the two ST8567 pairs at 0 and 72 h), these variable new alleles were 1.7–8.5 fold more likely to be encountered in strains isolated from a sample at 42 °C than from the same sample at 37 °C (Figure 2). The exception was the ST8567 strain pair at 7 d, which as discussed above was similar to the others in the total number of loci with new alleles but had an unusually low number of variable loci. In this pair, of the seven loci with variable new alleles, five were from 37 °C and just two from 42 °C (Figure 2).

**Figure 3:**
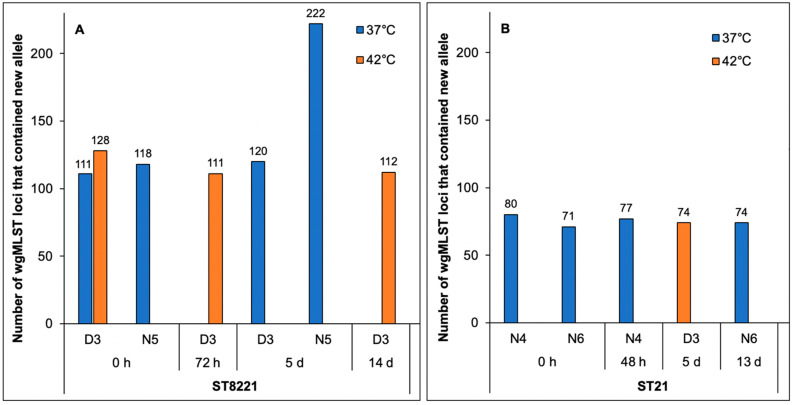
Incidence of wgMLST loci with new alleles in *C. jejuni* strains of (**A**) ST8221 and (**B**) ST21. Blue and orange indicate wgMLST loci with new alleles in strains isolated at 37 °C and 42 °C, respectively. New alleles include those that are the same in strains from 37 °C and 42 °C as well as those that vary between strains isolated at 37 °C vs. 42 °C. Analysis via wgMLST was carried out as described in Materials and Methods.

To further characterize the genomic diversity of strains isolated at 37 °C vs. 42 °C, we sequenced and analyzed via wgMLST several additional, unpaired strains. For these unpaired strains a sample yielded the indicated strain at one temperature, e.g., 37 °C, but we were unable to isolate a strain of the same ST from the same sample at the other temperature, e.g., 42 °C. These additional strains included five of ST8221 (three and two isolated at 37 °C and 42 °C, respectively) as well as five of ST21 (four and one isolated at 37 °C and 42 °C, respectively (Table 1 and Figure 3). Additional strains of ST8567 were not identified.

With one exception, the additional ST8221 genomes exhibited similar numbers of total new wgMLST alleles, ranging from 111 to 128 (Figure 3A). These numbers were also similar to those noted with the paired ST8221 strains discussed above (Figure 3A). The exception was a strain isolated at 37 °C 5 days post-treatment from animal N5, which exhibited a notably higher number (*n* = 222) of new alleles. This finding was unexpected, because a previous strain from the same animal at time 0 had only 118 new alleles, thus being similar to all others in regard to the incidence of new alleles (Figure 3A). The mechanisms underlying the higher number of new alleles in the strain isolated at 37 °C from this same animal five days later remain unidentified.

**Figure 4:**
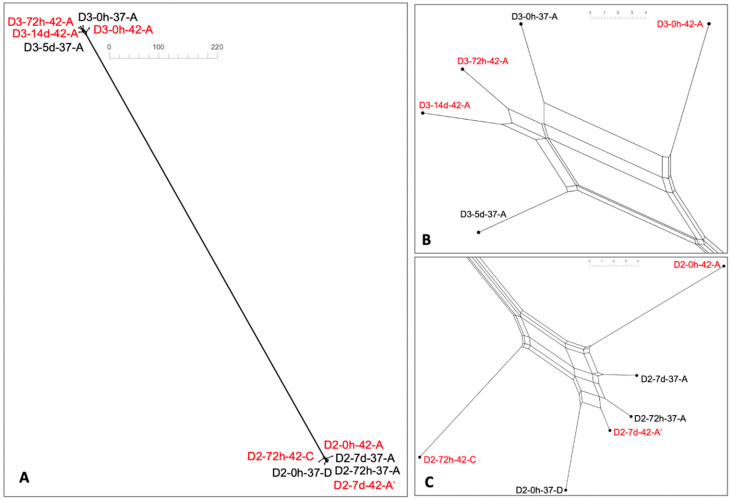
Neighbor-net SplitsTree for (**A**) paired ST8221 and ST8567, (**B**) zoomed-in ST8221, and (**C**) zoomed-in ST8567. Strains that were isolated at 42 °C are in red font. In SplitsTree, the distance between two strains corresponds to the sum of edge lengths of the shortest path between the two strains. Neighbor-net SplitsTree was obtained from wgMLST analysis as described in Materials and Methods.

As discussed earlier, most new alleles in the paired strains were shared between 37 °C and 42 °C and the number of shared alleles was similar for the strains in the pair, while new alleles that were only seen at one temperature were generally more numerous in the strain isolated at 42 °C (Figure 2). The same trend was noted when the three additional ST8221 strains were included in the analysis. The three ST8221 strains from steer D3 that were isolated at 42 °C had approximately 3-fold higher unique new alleles than the two strains from the same animal, and isolated at 37 °C (30 vs. 11) (Table S6). Similar findings were noted with the three ST8567 strains from steer D2 after isolation at 42 °C vs. the three strains isolated from steer D3 at 37 °C (33 vs. 11) (Table S6). This suggests that new alleles detected only at one temperature are more likely to be encountered in strains isolated at 42 °C. Neighbor-net SplitsTree analysis based on wgMLST showed that the five ST8221 strains were closely related, as were the six strains of ST8567, with a large distance between the two STs (Figure 4A). This agrees with the previous finding that ST8221 and ST8567 were both novel STs and only shared one allele in the seven-locus MLST scheme [9]. In spite of the close relatedness among the ST8221 strains, however, those isolated at 42 °C tended to exhibit higher variation (Figure 4B), with similar findings for the strains of ST8567 (Figure 4C).

The mechanisms underlying the observed higher prevalence of variable new alleles at 42 °C remain to be elucidated. The mutations that led to variable new alleles may have been pre-existing and conferred fitness advantages during the isolation of *C. jejuni* on mCCDA at 42 °C, thus promoting the establishment of the diversified alleles. It is important to keep in mind that in the course of isolation of *Campylobacter* from the bovine fecal samples, *C. jejuni* that may be present in the samples was first enriched through incubation at 37 °C in Bolton broth and subsequently transferred to the selective agar medium mCCDA that was incubated at either 37 °C or 42 °C. Thus, a temperature upshift may have been specifically experienced upon incubation at 42 °C on mCCDA, and the genomic changes observed at this temperature may have assisted the bacteria in their response to this elevated temperature. Previous studies have indeed shown a notable transcriptional response with induction of heat shock genes upon transition from 37 °C to 42 °C [11]. Such adaptive responses would not be needed when the bacteria were further incubated at 37 °C upon transfer to mCCDA. On the other hand, some of the variations that were noted at 37 °C may reflect adaptations of *C. jejuni* to growth at this temperature which, albeit fully permissive of growth, may still be sensed by the bacteria as suboptimal. *C. jejuni* has impressive thermosensing and transcriptional response capacities that can efficiently differentiate between 37 °C and 42 °C [11,18]. For instance, the transcriptional repressor HrcA, critical in mediating *C. jejuni*’s heat-shock response, irreversibly loses its DNA-binding activity upon transition from 37 °C to 42 °C [18]. It is also possible that the higher growth rate of *C. jejuni* at 42 °C [11,19,20] may increase the frequency of mutations at this temperature.

Besides the two novel STs 8221 and 8567, the three dominant *C. jejuni* STs from the feces of the cattle in our ceftiofur study included ST21, which belongs to the same clonal complex (CC21) as ST8567 [9]. Analysis of the five available ST21 genomes (Table 1) revealed that the number of loci with new alleles was similar between strains from different temperatures (ranging from 71 to 80) (Figure 3B) and lower than noted with ST8567 for which new alleles ranged from 111 to 122 (Figure 2). The lower number of loci with new alleles in ST21 than ST8567 may reflect the fact that ST21 is a long-known and widely-encountered ST [21], with much of its allelic variation already identified.

As noted above with the paired genomes, most temperature-variable loci involved new alleles (Table S2). Among the relatively few variable loci involving existing alleles, CAMP1331 (Cj1420c) encoding a putative methyltransferase was found to be variable in all three STs while CAMP0157 (Cj0170) encoding the hypothetical protein Cj0170 was variable in both ST21 and ST8221 (Table S2).

### 2.3. Missense and Frameshift Mutations Predominated among the Variable Loci

Amino acid substitutions mediated by missense mutations were noted in over half of the variable loci in most strain pairs, followed by frameshift mutations that mostly (20/21) led to the emergence of premature stop codons (PMSCs) (Table 2 and Table S3). Because the original vs. variant allele in the missense and silent substitutions could not be differentiated, the impact of temperature related to these two types of mutations could not be determined. In the case of loci with alleles harboring frameshift, nonsense, and start-loss mutations likely to lead to non-functional polypeptides, the allele harboring such mutations was considered to be a variant of the original wildtype allele, and we analyzed the frequency of such mutations in strains isolated at 37 °C vs. 42 °C.

More loci with frameshift, nonsense, and start-loss mutations were found at 42 °C than 37 °C (19 vs. 8), with primarily new alleles, and the variation was largely localized in homopolymeric tracts (HTs) (Table 2 and Table S3). Most variable loci with frameshift, nonsense, and start-loss mutations were only found via wgMLST analysis, except for nine cgMLST loci (CAMP0341, CAMP1214, CAMP1261, CAMP0260, CAMP0028, CAMP1605, CAMP0342, CAMP0912, and CAMP0926). Furthermore, most (13/15) of the loci with nonsense and start-loss mutations were found at 42 °C (Table 2 and Table S3).

About half (12/25) of the frameshift and nonsense mutations were in the first 50.7% of the coding sequence and 9 of the 12 variable loci with such mutations were exclusively identified among strains isolated at 42 °C (Table S3), suggesting potential selection for loss-of-function mutations at this temperature. It is also noteworthy that CAMP0575 (hypothetical protein Cj0617) and CAMP0912 (putative membrane protein) harbored frameshift and start-loss mutations in both ST 8221 and 8567 (Table 2). These repeated occurrences suggest that these loci may have a propensity to alter their original functions. In strain pairs of both ST8221 and ST8567, the frameshift in CAMP0575 that involved insertions in HTs occurred at 37 °C, while the start-loss mutations in CAMP0912 occurred at 42 °C (Table 2 and Table S3). Such findings suggest that loss of function of these genes may be favored at different temperatures. Besides CAMP0575 and CAMP0912, CAMP0913 (hypothetical protein Cj0990c) was also found to harbor missense mutations in both ST 8221 and 8567, though no temperature preference was observed (Table 2 and Table S3).

Analysis of the variable loci revealed some loci that appear to be duplicated or paralogous. Specifically, in the ST8567 strain pair at 7 d the new alleles in CAMP1236 and CAMP1252 (related to motility) were the same, as were the previously known alleles at CAMP1651 and CAMP1655, of unknown function (Table S3). PubMLST analysis revealed additional known alleles that were identical between CAMP1651 and CAMP1655 (data not shown). Thus, CAMP1236 and CAMP1252 as well as CAMP1651 and CAMP1655 may represent duplicated or paralogous loci.

### 2.4. Variation in HTs Is Predominant among the Variable Loci

Of the 75 variable loci from the strain pairs of ST8221 and ST8567, 50 involved variations in HTs, primarily poly G, poly A, and poly T (Table S3). Several of the loci with variation in HTs putatively encoded membrane proteins (*n* = 5; CAMP0279, CAMP0480, CAMP0545, CAMP0608, and CAMP0926), periplasmic proteins (*n* = 3), iron binding (*n* = 3), and methyltransferase (*n* = 3), while others were mainly implicated in motility (*n* = 4), DNA/RNA metabolism (*n* = 3), and metabolism (*n* = 2) (Table S3). The motility-related genes include CAMP0040, CAMP1236, CAMP1252, and CAMP1258, with CAMP1258 found to be variable more than once in ST8567 (Table S3). Several (17/75) of the variable loci were previously found to be prone to variation in *C. jejuni* from other sources, including milk and water-borne outbreaks, human cases, and experimental murine infections [17,22,23,24,25,26,27] (Table S4). Several of these 17 previously reported loci exhibited variation in HTs in more than one study, and encoded phase-variable genes [26]. For example, CAMP1214, CAMP1243, and CAMP1258 are in the flagellum biosynthesis region of *C. jejuni* NCTC 11168 [26], CAMP1331 is a methyltransferase located in the capsule biosynthesis locus [26], and CAMP0631 regulates the surface-associated invasion gene *cipA* [26,28]. It is noteworthy that six loci found to be variable in our study (CAMP0019, CAMP0608, CAMP0631, CAMP1214, CAMP1243, CAMP1258, and CAMP1331) were also found to vary among isolates from dairy cows, patients, and the milk tank in a milk-borne outbreak [26]. Taken collectively, the available data suggest that the phase variation in these genes may not only reflect a response to temperature during isolation on the selective media but may have other important adaptive roles. Such phase variation may, for instance, assist *C. jejuni* to rapidly adapt to the surrounding environment [26,29] and colonize different hosts [17,22,23,24,25,27].

### 2.5. Certain Variable Loci Were Encountered in Strain Pairs from Different Time Points

The three strain pairs of ST8567 were obtained at different times from the same animal, allowing us to assess persistence of the observed temperature-associated variation. Of the 44 loci of ST8567 found to be variable by temperature, four, i.e., CAMP0044 (putative iron-binding protein), CAMP1243 (putative methyltransferase), CAMP1258 (motility accessory factor), and CAMP1644 (hypothetical protein) were found to be variable at multiple time points (Table 2 and Table S3). In all four loci, variation involved one-two nucleotide insertions or deletions (indels) in polyG repeats resulting in premature stop codons (PMSCs) shortly after the indel (Table S3). The nucleotide indels were at the same location in different ST8567 strains (Table S3), suggesting that the findings are unlikely to be caused by sequencing error. The frameshifts were identified at both temperatures in ST8567 (Table S3). For instance, a G deletion in CAMP0044 (after nt 710) was detected twice at 37 °C (0 and 72 h), as well as at 42 °C (7 d), while the indel of G or GG (around nt 249) was detected at 42 °C (0 h), 37 °C (72 h), and both temperatures (7 d) (Table S3). It was noteworthy that on two separate occasions (0 h and 7 d) G insertions or deletions in polyG repeats were detected in three of these four loci, and on both occasions the strains were isolated at 42 °C (Table S3). Loci CAMP0044, CAMP1243, CAMP1258, and CAMP1644 were found to be variable at multiple time points, suggesting a general selection for frameshifts in polyG repeats that resulted in PMSCs. However, the small number of genomes prevents us from assessing the extent to which insertions or deletions at the polyG repeats at these loci are selected upon under different temperatures. The four loci were identified only by wgMLST analysis, indicating the necessity of performing wgMLST to complement cgMLST.

### 2.6. Directions for Future Research

Currently we lack information on the potential impact of isolation temperature (37 °C vs. 42 °C) on the genome sequence of *C. jejuni*. In this study we pursued a comparative analysis of the genomes of several strain pairs isolated from bovine fecal samples upon cultivation on selective agar media at both 37 °C and 42 °C, including one strain pair of ST8221 and three of ST8567, and discovered limited but consistent variation between strains isolated at different temperatures. In all but one of the strain pairs, variable loci harboring new alleles were more likely to be encountered upon isolation at 42 °C, suggesting that this isolation temperature may favor genomic diversification. Furthermore, most (9/12) frameshift and nonsense mutations that were in the first half of the coding sequence were detected at 42 °C, which thus may select for loss-of-function mutations. Loci such as CAMP0575, CAMP0912, and CAMP0913 were found to be variable by temperature in strains of both ST8221 and ST8567, suggesting that they may be especially prone to diversification in response to isolation temperature. Such findings suggest that the incubation temperature for isolation of *C. jejuni* on selective media such as mCCDA may impact the genome sequence, and would need to be taken into account in studies of the molecular epidemiology of *C. jejuni* in bovine reservoirs and in assessment of relevant food safety-related mitigations. Future research employing functional assays is needed to reveal whether the mutations confer specific fitness advantages to *C. jejuni* during isolation on selective media at 42 °C.

A noticeably higher number of variable loci was identified by cg/wgMLST analysis than by the SNP cluster analysis employed by the NCBI pipeline (Table S5). The NCBI pipeline assigned all strains of ST8221 and ST8567 to SNP clusters PDS000091235.1 and PDS000091234.1, respectively (Table 1). The paired ST8221 strains at 0 h only exhibited 2 SNPs in contrast to 25 wgMLST allele differences, while the ST8567 strain pairs at 0 h and 72 h had only three or four SNPs, again lower than observed with wgMLST analysis (Figure 4 and Table S5). The different number of variable loci detected by cg/wgMLST and SNP cluster analysis may reflect the different methodology and algorithms for these analyses. Another study also reported a noticeably higher number of variable wgMLST loci revealed by Genome Comparator than by SNP analysis via read mapping (35 vs. 9) in *C. jejuni* of ST53 [30]. More variation via wgMLST analysis than via SNP analysis was also noted with *Listeria monocytogenes* [31]. The strains discussed above belonged to unique SNP clusters and closely-related reference genomes were not identified. Therefore, employing Genome Comparator in this study is more appropriate as it does not require a closely-related reference genome, compared to the reference-based SNP analysis. Our study employed specific STs of *C. jejuni* from bovine fecal samples, and further research is needed to determine whether similar trends may be identified with other STs of *C. jejuni*. Future studies are also needed to assess the extent to which isolation temperature may impact genome content in other *Campylobacter* species frequently isolated from cattle and other animals, such as *C. coli*.

## 3. Materials and Methods

### 3.1. C. jejuni Strains and Culture Conditions

The 18 *C. jejuni* strains that were selected to examine the temperature impact on the genome sequence were isolated during our previous study that assessed the effect of ceftiofur treatment of steers on *Campylobacter* [9]. All strains were isolated as previously described by selective enrichments of the fecal samples in Bolton broth at 37 °C following by plating of 0.1 mL of the enrichment on mCCDA plates that were incubated at 37 °C or 42 °C [9]. The strain panel included eight paired strains, i.e., one strain that had been isolated on mCCDA at 37 °C and another isolated on mCCDA at 42 °C from each of the four fecal samples. The paired strains included two of ST8221 from a fecal sample collected pre-treatment from steer D3, and six strains of ST8567 from steer D2. Two of these paired ST8567 strains were from a pre-treatment sample, while the other four were from two different post-treatment samples (Table 1). In addition to the paired strains, we included in the analysis five strains of ST8221 and five of ST21, obtained at 37 °C or 42 °C (Table 1). 

### 3.2. Whole Genome Sequencing (WGS), Assembly, and Annotation

*C. jejuni* was grown at 42 °C overnight and DNA extracted as previously described using a Qiagen DNeasy blood and tissue kit (Qiagen, Gaithersburg, MD, USA) [32]. Sequencing libraries were prepared using a Nextera XT DNA sample preparation kit (Illumina, Inc., San Diego, CA, USA), sequenced following a 300 bp paired-end protocol using Illumina MiSeq (Illumina), and the produced sequencing reads were demultiplexed using MiSeq reporter software (Illumina). Both DNA extraction and sequencing were based on manufacturer’s instructions. Depth of coverage of ≥30 (30×) was applied to assure sequence quality and genomes with less than 30× sequence coverage were re-sequenced.

The assembly of genomes was performed using SPAdes 3.13.0 with internal mismatch correction turned on using the “--careful” option under default settings [33]. QUAST was used to assess the quality of all stringent assemblies (http://cab.cc.spbu.ru/quast/ (accessed on 22 October 2020), including sequence size, number of contigs, N50, and L50 (Table S1). The number of reads and average read length were determined using CLC Genomics Workbench, version 11.0 (CLC bio, Germantown, MD, USA) (Table S1). The assembled sequences were uploaded to the Rapid Annotation using Subsystem Technology Server (RAST) [34] and then annotated by RAST [35]. Mean sequence size was determined based on contig statistics from RAST (Table S1).

### 3.3. cgMLST, wgMLST, and Single-Nucleotide Polymorphism (SNP) Cluster Analysis

cgMLST and wgMLST data were analyzed to determine allele differences using the *Campylobacter jejuni*/*coli* Genome Comparator [36], with the *C. jejuni*/*C. coli* cgMLST v1.0 (1343 loci) scheme and the wgMLST scheme with all CAMP loci (1970 loci). The combined cgMLST variable loci and wgMLST variable loci are referred to in this work as “variable loci”, unless otherwise specified. Loci that contained missing or incomplete alleles were excluded from the analysis. To locate the position of variant nucleotides, the commands “Produce alignments” and “Create alignment stats” were selected under “Alignments” in Genome Comparator [36]. Additionally, the Neighbor-net SplitsTree that was used to visualize the distance between any two strains was generated by Genome Comparator. To investigate the impact of variable loci on the amino acid (AA) sequences, the allele sequences derived from PubMLST Alignments were aligned using PATRIC 3.6.9 by selecting the Multiple Sequence Alignment, Amino Acid option [37]. The allele sequence and the deduced polypeptide sequence were also examined using RAST [34]. The number of isolates that contained an existing allele that caused a frameshift was determined using the *Campylobacter jejuni*/*coli* typing database (https://pubmlst.org/bigsdb?db=pubmlst_campylobacter_seqdef (accessed on 15 October 2020). To identify SNP clusters, we employed the NCBI Pathogen Detection Pipeline (https://www.ncbi.nlm.nih.gov/pathogens/ (accessed on 16 June 2021) using the accession numbers listed in Table 1.

### 3.4. Data Availability

The WGS data analyzed in this study were from BioProject PRJNA727200 (BioSamples SAMN19009700 to SAMN19009706 and SAMN19009708; Table 1) and have been deposited at the NCBI Sequence Read Archive (SRA) database. The SRA accession numbers were shown in Table 1.

## Figures and Tables

**Table 1:** *C. jejuni* strains investigated in this study.

Strain	Temperature (°C)	ST ^3^	Steer	Time ^4^	SNP Cluster	Accession	BioSample	SRA Accession
D3-0h-37-A ^1^	37	8221	D3	0 h	PDS000091235.1	PDT001064031.1	SAMN19009706	SRS8893461
D3-0h-42-A ^1^	42	8221	D3	0 h	PDS000091235.1	PDT001064029.1	SAMN19009708	SRS8893463
N5-0h-37-A	37	8221	N5	0 h	PDS000091235.1	PDT001064064.1	SAMN19009714	SRS8893428
D3-72h-42-A	42	8221	D3	72 h	PDS000091235.1	PDT001064028.1	SAMN19009709	SRS8893464
D3-5d-37-A	37	8221	D3	5 d	PDS000091235.1	PDT001064030.1	SAMN19009707	SRS8893462
N5-5d-37-B	37	8221	N5	5 d	PDS000091235.1	PDT001064063.1	SAMN19009715	SRS8893429
D3-14d-42-A	42	8221	D3	14 d	PDS000091235.1	PDT001064067.1	SAMN19009711	SRS8893426
D2-0h-37-D ^2^	37	8567	D2	0 h	PDS000091234.1	PDT001064070.1	SAMN19009700	SRS8893422
D2-0h-42-A ^2^	42	8567	D2	0 h	PDS000091234.1	PDT001064047.1	SAMN19009703	SRS8893445
D2-72h-37-A ^2^	37	8567	D2	72 h	PDS000091234.1	PDT001064069.1	SAMN19009701	SRS8893423
D2-72h-42-C ^2^	42	8567	D2	72 h	PDS000091234.1	PDT001064036.1	SAMN19009704	SRS8893456
D2-7d-37-A ^2^	37	8567	D2	7 d	PDS000091234.1	PDT001064058.1	SAMN19009702	SRS8893434
D2-7d-42-A’ ^2^	42	8567	D2	7 d	PDS000091234.1	PDT001064032.1	SAMN19009705	SRS8893460
N4-0h-37-A	37	21	N4	0 h	PDS000091232.1	PDT001064066.1	SAMN19009712	SRS8893425
N6-0h-37-A	37	21	N6	0 h	PDS000091232.1	PDT001064062.1	SAMN19009716	SRS8893430
N4-48h-37-C	37	21	N4	48 h	PDS000091232.1	PDT001064065.1	SAMN19009713	SRS8893427
D3-5d-42-A	42	21	D3	5 d	PDS000091232.1	PDT001064068.1	SAMN19009710	SRS8893424
N6-13d-37-A’	37	21	N6	13 d	PDS000091232.1	PDT001064061.1	SAMN19009717	SRS8893431

^1^ Paired strains of ST8221 from a fecal sample of steer D3. ^2^ Paired strains of ST8567 from three different fecal samples of steer D2. ^3^ ST21 and ST8567 belong to clonal complex (CC) 21; ST8221 belongs to CC61. ^4^ Time related to ceftiofur treatment, with 0 h corresponding to immediately prior to treatment and other times being hours (h) or days (d) after treatment.

**Table 2:** Impacts of variable loci (VL) on the deduced polypeptide sequences based on analysis via Genome Comparator.

Strain Pair	Number of VL	Impacts on the Deduced Polypeptide Sequences							
		Missense	Silent	Frameshift		Nonsense		Start-Loss	
		Number of VL ^1^	Number of VL ^1^	Number of VL ^1^	Locus ^1, 2, 3^	Number of VL ^1^	Locus ^1, 2^	Number of VL ^1^	Locus ^1, 2, 3^
ST8221 0 h ^4^	25	15	1	2 (1L, 1**H**)	CAMP0341, 5A, **H** CAMP0575, 10G, L	3 (3**H**)	CAMP0028, **H** CAMP1605, 8A, **H** CAMP1680, **H**	3 (3**H**)	CAMP0342, **H** CAMP0912, **H** CAMP0926, 6T, **H**
ST8567 0 h	24	14	1	10 (3**L**, 5**H**, 1H, 1**HL**)	CAMP1214, 11G, **HL** CAMP1261, 8A, **H** CAMP0044, 10G, **L** CAMP0575, 11G, **L** CAMP1223 ^4^, 11G, **H** CAMP1228, 9G, **H** CAMP1243, 11G, **H** CAMP1258, 10G, **H** CAMP1644, 9G, H CAMP1649, 10G, **L**	0	0	1 (1**H**)	CAMP0912, **H**
ST8567 72 h ^4^	19	11	5	5 (2**L**, 1L, 1**H**, 1H)	CAMP0260, 9T, **L** CAMP0044, 10G, **L** CAMP0827, **H** CAMP1331, 10G, H CAMP1644, 10G, L	1 (1**H**)	CAMP0345, 5A, **H**	0	0
ST8567 7 d	7	3	0	4 (1**L**, 2**H**, 1HL)	CAMP0044, 10G, **H** CAMP1243, 10G, **L** CAMP1258, 9G, **H** CAMP1644, 10G, HL	0	0	0	0

^1^ Bold: variation that was caused by new allele at 37 °C (L), 42 °C (H), or both temperatures (HL). Locus noted with HL is excluded when comparing the number of loci at either temperature. The number of variable loci involving new or existing allele at the corresponded temperature is indicated in parentheses under “Number of variable loci”. ^2^ The homopolymeric tract is denoted by the number of nucleotide repeats. ^3^ Underlined: six variable loci that resulted in frameshift (CAMP0044, CAMP0575, CAMP1243, CAMP1258, and CAMP1644) or start-loss mutations (CAMP0912) in more than one strain pair. ^4^ CAMP0631 in ST8221 0 h strain pair was not included due to nucleotide substitution in an intergenic region; CAMP1223 in ST8567 0 h strain pair resulted in missense, silent substitution, and frameshift mutations; CAMP1433, CAMP1236 and CAMP1252 in ST8567 72 h strain pair resulted in missense and silent substitution mutations.

## Data Availability

The WGS data analyzed in this study were deposited in NCBI BioProject Accession PRJNA727200 (BioSamples SAMN19009700 to SAMN19009717).

## Supplementary Materials

The following supporting information can be downloaded at: https://www.mdpi.com/article/10.3390/pathogens11060678/s1, Table S1: Whole genome sequencing (WGS) information of *C. jejuni* strains investigated in this study; Table S2: Complete output of cg/wgMLST analysis; Table S3: Loci found to be variable loci between strains of ST8221 and ST8567; Table S4: Variable loci that were reported in this study and other studies; Table S5: Single-nucleotide polymorphism (SNP) cluster analysis; Table S6: Unique alleles of ST8221 and ST8567 strains isolated at different temperatures.
